# Predictors of acute upper GI toxicity and its correlation with dosimetric analysis in patients receiving adjuvant radiation therapy for breast cancer

**DOI:** 10.3332/ecancer.2025.2020

**Published:** 2025-10-22

**Authors:** Tooba Ali, Nasir Ali, Syed Aun Hasan, Nawazish Zehra, Laraib Khan, Maham Khan, Mariam Hina, Bilal Ahmed, Fabiha Shakeel, Ahmed Nadeem Abbasi, Bilal Mazhar Qureshi, Asim Hafiz, Maria Tariq, Sehrish Abrar

**Affiliations:** 1 Department of Oncology Section Radiation Oncology, Aga Khan University, Karachi 74800, Pakistan; 2 Department of Oncology, Sindh Institute of Urology and Transplant, Karachi 74200, Pakistan

**Keywords:** breast radiation, radiation-induced nausea vomiting, quality of life, VMAT

## Abstract

**Introduction:**

Breast cancer is the most common malignancy in women worldwide, with significant geographic variation in incidence. Adjuvant radiotherapy (RT) improves outcomes but can cause underrecognised toxicities such as radiation-induced nausea, vomiting (RINV) and esophagitis. This study aims to correlate RINV and esophagitis with organ-at-risk (OAR) dose constraints and clinical factors.

**Materials and method:**

This prospective observational study analysed upper gastrointestinal (GI) toxicity in breast cancer patients receiving adjuvant RT. Patient demographics, clinical characteristics and dose-volume parameters for the stomach and esophagus were recorded. Statistical analyses identified predictors of RINV and esophagitis using univariate and multivariate models. Significant correlations were reported with a *p*-value <0.05.

**Results:**

The study included 110 female breast cancer patients (mean age 51 years), with volumetric modulated arc therapy (VMAT) as the predominant radiation technique and moderate hypofractionation (42.56 Gy/16 fractions) as the most prescribed regimen. Acute upper GI toxicity was observed in 60.9% of patients, with 3.6% experiencing Grade 3 toxicity. Higher stomach dose exposure (D10, D30 and D60cc) significantly correlated with increased toxicity (*p* < 0.001). Free breathing was associated with a higher risk of GI toxicity compared to deep inspiration breath-hold (DIBH) (*p* = 0.035), which showed a protective effect. Dysphagia was reported in 70% of patients, with esophageal mean dose strongly predicting severity (*p* < 0.001). Multivariate analysis confirmed stomach D30 cc and esophageal mean dose as independent predictors of high-grade toxicity. VMAT was associated with slightly higher toxicity compared to three-dimensional conformal RT.

**Conclusion:**

Radiation technique, dose-volume parameters and breathing strategy significantly influence acute upper GI toxicity and dysphagia in breast cancer RT. DIBH reduces GI toxicity risk, while higher stomach and esophageal doses correlate with increased toxicity. Incorporating the stomach as an OAR and optimising treatment planning can enhance tolerability and improve patient outcomes.

## Introduction

Breast cancer is the most common malignancy among women and ranks as the second most common cancer worldwide [1]. Its incidence varies geographically, with significant differences between regions [2]. According to GLOBOCAN estimates, the incidence rate per 100,000 is 74.1 in developed regions and 31.3 in less developed areas, while in South-Central Asia, it stands at 28.2 per 100,000 [3]. In Pakistan, one in nine women is at risk of developing breast cancer during her lifetime [4].

Depending on the stage and clinical scenario, the treatment of breast cancer typically involves a multidisciplinary approach incorporating surgery, chemotherapy, radiation therapy, endocrine therapy and targeted therapy [5]. The beneficial effect of radiation therapy in the adjuvant setting is well-established. Several meta-analyses have demonstrated that adding radiation therapy after surgery significantly improves local control and prolongs disease-free survival [6].

Radiotherapy (RT) fractionation regimens have evolved, ranging from standard fractionation (50 Gy in 25 fractions) to hypofractionation (42.5 Gy in 16 fractions) and ultra-hypofractionation (26 Gy in 5 fractions) [5]. Advances in radiation delivery techniques have also enhanced treatment precision, ensuring dose homogeneity to the target tissue while minimising radiation-induced toxicity to surrounding organs. These modern techniques include three-dimensional conformal RT (3DCRT), intensity-modulated radiation therapy (IMRT) and volumetric modulated arc therapy (VMAT) [7, 8].

Despite these advancements, adjacent organs at risk (OAR) still receive a fraction of the daily delivered dose. According to the Multinational Association of Supportive Care in Cancer (MASCC), the emetogenic risk of radiation to the breast is less than 30% [9]. However, Lazzari *et al* [10] reported that approximately 64% of patients treated for left-sided breast cancer experienced acute radiation-induced nausea and vomiting (RINV) [10]. RINV is primarily attributed to irradiation of OARs in the upper abdomen, particularly through stimulation of the peripheral emetic trigger zone. Since these OARs are located deep within the abdomen and generally receive lower radiation doses, RINV in breast cancer patients has been historically overlooked [11].

A pilot interview-based study has provided valuable insights into patients' experiences with RINV. It emphasised the importance of defining nausea from the patient's perspective, as the absence of a consistent definition in prior literature has likely contributed to variability in reported outcomes [12]. Radiation-induced esophagitis (RE) is another common side effect, particularly among patients receiving radiation to the supraclavicular region [13]. A 2021 study reported that over 15% of patients undergoing RT for breast and supraclavicular regions experienced grade II esophagitis/dysphagia [13].

Unfortunately, these side effects remain underreported and underrecognised in breast cancer RT.

In light of such disparities in previously reported literature, this study was designed with the primary objective of correlating the incidence of RINV during adjuvant radiation therapy for breast cancer with OAR dose constraints and patients' characteristics, the secondary objective is to determine the incidence of RE and identify dose-volume predictors and clinical parameters in patients undergoing adjuvant radiation to breast/chest wall with or without regional nodal irradiation (RNI).

## Materials and methods

### Ethics approval

This study was conducted following approval from the Ethics Review Committee at our institution.

### Study design and cohort

This was a prospective observational study conducted between July and December 2024 that included all patients undergoing adjuvant radiation to the breast or chest wall with or without RNI. This study was conducted in accordance with the Strengthening the Reporting of Observational Studies in Epidemiology reporting guidelines.

### Radiation therapy planning and treatment

All patients mandating indications for adjuvant radiation with various regimens of hypofractionation were included [14]. Each patient was planned for simulation in supine position on Vac lock (vacuum mat) with both arms abducted and positioned above the head. For left breast/chest wall irradiation patients were tutored with the deep inspiration breath hold (DIBH) technique, patients unable to perform DIBH proceeded with free breathing (FB) radiation planning. The RT planning computed tomography (CT) scans were imported into the planning software, and target volumes were delineated according to established contouring guidelines [15]. These included the clinical target volume for the primary site and regional lymph nodes, based on the Radiation Therapy Oncology Group (RTOG) recommendations. The supraclavicular fossa was contoured following the RTOG breast cancer atlas, extending cranially from the inferior border of the cricoid cartilage to the caudal edge of the clavicle. A setup error margin was added to define the planning target volume (PTV). OARs were also contoured, including the esophagus (from just below the cricoid cartilage to the aortic notch) and the stomach (from the gastroesophageal junction to the pylorus).

Radiation plan evaluation and approval were done by the primary radiation oncologist. During target volume evaluation, dose volume histogram (DVH) for OARs was also analysed, with emphasis on mean doses and percentage of volume receiving doses at various points on the histogram. All patients were treated in the supine position, and upper gastrointestinal (GI) toxicity and esophagitis experienced by each patient were assessed and documented weekly in chart reviews as part of general radiation-associated toxicity management.

Data collection was conducted through the radiation oncology information system, Adaptive RT Integrated Algorithm, along with weekly reviews of patients' charts and electronic files. Patients receiving adjuvant RT to the breast/chest wall with or without RNI were included. Patients' demographics and clinical characteristics, including age, body mass index (BMI), tumor, node, metastasis, staging, hormone receptor status, type of fractionation regimen prescribed, volume treated and techniques 3DCRT, IMRT or VMAT used were recorded. A comparative analysis of acute upper GI toxicity outcomes across these techniques was planned. The side effect profile of patients was ascertained from weekly chart reviews done during the treatment process as per the RTOG scale for upper GI toxicity (nausea and vomiting) and dysphagia scale. Exclusion criteria for the study included male breast cancer, metastatic disease and individuals with a known history of upper GI disorders, such as peptic ulcer disease, dysphagia or esophageal motility disorders as these conditions may complicate the assessment process and their inclusion could confound the study outcomes.

DVH doses of esophagus and stomach were documented, these include D 60 cc (60 cc volume of stomach receiving particular dose), D 30 cc (30 cc volume of stomach receiving particular dose) and D 10 cc (10 cc of volume receiving particular dose) for stomach and subset of patient receiving radiation therapy to supra clavicular area mean dose to esophagus were recorded. These recorded values were then correlated with acute upper GI toxicity and dysphagia reported by patients during weekly reviews throughout the course of treatment.

As part of the standard RT consent process, patients provided permission for their data to be used for research purposes.

### Statistical analysis

Data were analysed using SPSS version 23.0. Descriptive statistics summarised patient demographics, tumour characteristics and treatment parameters. Associations between acute upper GI toxicity, dysphagia and radiation treatment characteristics were analysed using univariate tests, including chi-square, Independent sample *t*-tests and Mann-Whitney *U* tests. Categorical data were analysed using the Wilcoxon test to evaluate differences between independent groups. Linear regression models were applied for both univariate and multivariate analyses to examine the relationship between variables. Spearman's and Pearson's correlation coefficients were calculated to assess the strength and direction of the associations between variables. Subgroup comparisons between radiation techniques (3DCRT, IMRT and VMAT) and toxicity outcomes were performed using *post hoc* analysis. Multivariate logistic regression identified independent predictors of high-grade upper GI toxicity and dysphagia, incorporating variables such as stomach and esophageal doses, radiation technique, breathing technique (DIBH versus FB) and clinical parameters. Significant predictors were reported with odds ratios and 95% confidence intervals. A *p*-value <0.05 was considered statistically significant.

## Results

The study included 110 female patients with a mean age of 51 (Range 29–72) years. The most common histopathological diagnosis was invasive ductal carcinoma. The chest wall, axilla and supraclavicular region were the most frequently irradiated sites. Most patients had a BMI greater than 30. VMAT was the predominant radiation technique utilised. The most administered radiation dose was moderate hypofractionation (42.56 Gy in 16 fractions at 2.66 Gy per fraction). Dosimetric data from all 110 CT planning scans and treatment plans were evaluated. A summary of the overall treatment characteristics of the study cohort is presented in Table 1.

### Correlation between acute upper GI toxicity and radiation treatment characteristics

**Figure 1.**
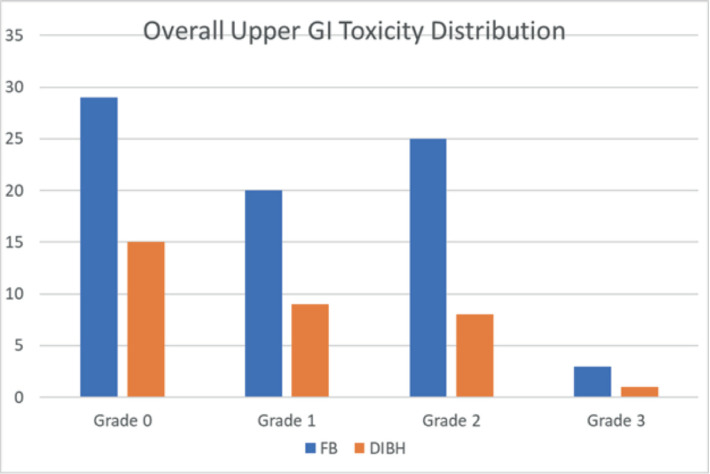
Overall upper GI toxicity distribution in each cohort.

In our study, 33 patients were included in the DIBH cohort and 77 in the FB cohort. Regarding upper GI toxicity (Table 2), 29 patients (26.3%) experienced Grade 1 toxicity (mild symptoms with <5% weight loss), while 33 patients (30%) developed Grade 2 toxicity (moderate symptoms with <15% weight loss, requiring antiemetics). Severe toxicity (Grade 3), characterised by anorexia and weight loss exceeding 15%, was observed in 4 patients (3.6%). Figure 1 illustrates the overall distribution of upper GI toxicity grades across both cohorts.

**Figure 2.**
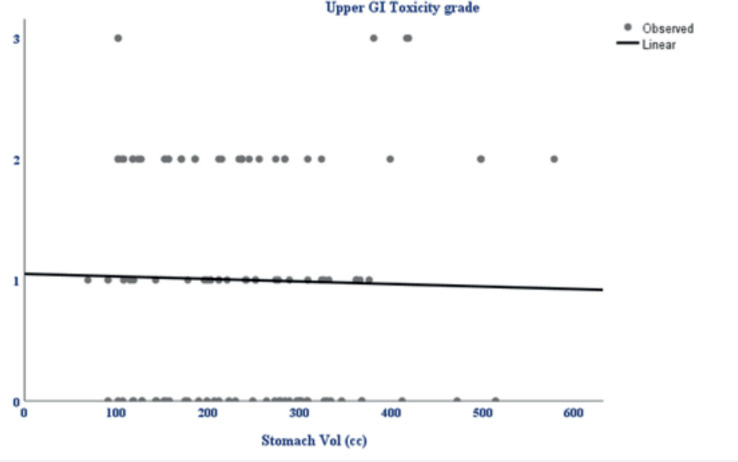
Scatterplot showing the relationship between stomach volume (cc) and upper GI toxicity grade. The linear trendline indicates a weak negative correlation, suggesting no significant association between increasing stomach volume and upper GI toxicity grade.

In the univariate analysis, (Table 3) several variables were assessed for their association with acute upper GI toxicity. No statistically significant relationship was found between acute upper GI toxicity and hormonal receptor status (*p* = 0.18), type of surgery (*p* = 0.072), BMI (*p* = 1.00) or stomach volume (*p* = 0.8) (Figure 2). However, the radiation technique was significantly associated with the development of acute upper GI toxicity (*p* = 0.010) and higher RT dose delivered (boost) was significantly associated with acute upper GI toxicity (*p* = 0.026). Moreover, a strong positive correlation was observed between higher fractionated dose delivered to D10 cc (*p* = 0.0001), D30 cc (*p* = 0.0001) and D60 cc (*p* = 0.0001) of stomach and acute upper GI toxicity, indicating that increased stomach dose exposure is associated with higher toxicity risk.

Overall, the significant factors influencing acute upper GI toxicity included radiation technique, RT dose and dose to stomach (D10 cc, D30 cc and D60 cc). Importantly, left chest wall or breast irradiation with or without RNI during FB was associated with a significantly higher risk of GI toxicity compared to DIBH, which holds a significant negative correlation implying a protective effect and helps in reducing GI toxicity (*p* = 0.035) (Supplementary

Figures 1a,b and 2a,b). Additionally, a positive correlation was found between larger PTV volume and the risk of developing acute upper GI toxicity (*p* = 0.036).

Further subgroup analysis using *post hoc* testing evaluated the association between radiation technique and upper GI toxicity, with significant differences observed among certain radiation techniques. Notably, patients treated with VMAT experienced slightly higher upper GI toxicity compared to those treated with 3DCRT with a mean difference of −0.815 (*p* = 0.000).

In contrast, no statistically significant difference was found in upper GI toxicity between 3DCRT and IMRT (*p* = 0.882) or between VMAT and IMRT (*p* = 0.083), indicating that IMRT does not significantly alter the toxicity profile compared to either 3DCRT or VMAT (Table 4) (Supplementary Figure 3a and b).

A subgroup analysis was conducted to assess the association between RT dose and acute upper GI toxicity. Patients were divided into two groups based on the prescribed RT dose: those who received a boost to the lumpectomy cavity/chest wall scar and those who did not. The mean upper GI toxicity grade was 1.12 (95% CI: 0.85–1.39) in the low-dose group compared to 0.90 (95% CI: 0.67–1.13) in the high-dose group. The standard deviation of toxicity grades was 0.949 for the low-dose group and 0.926 for the high-dose group, indicating a similar variability in toxicity levels across both groups.

An independent samples *t*-test was performed to determine whether the difference in mean toxicity grades between the two groups was statistically significant. The *t*-test showed no significant difference (*p* = 0.153), suggesting that RT doses alone may not be an independent predictor of acute upper GI toxicity. Subgroup analysis revealed no significant difference in upper GI toxicity grades between patients who received a boost and those who did not (*p* = 0.153).

A crosstabulation analysis was performed to explore the association between volume irradiated and upper GI toxicity grade. Irradiated volumes were categorised into three subgroups: <500 cc, 500–1,500 cc and >1,500 cc. Among patients with Grade 1 toxicity, 65.5% belonged to the 500–1,500 cc group, while 27.6% and 6.9% were in the >1,500 and <500 cc groups, respectively. For patients with Grade 2 toxicity, the majority (87.9%) had 500–1,500 cc of volume irradiated, whereas 9.1% were in the >1,500 cc group and 3.0% in the <500 cc group. Among patients with Grade 3 toxicity, 60% were in the 500–1,500 cc group and 40% in the >1,500 cc group, with no cases observed in the <500 cc group. Overall, higher grades of GI toxicity (Grades 2 and 3) were more frequently seen in patients with larger irradiated volumes (>1,500 cc) compared to those with smaller volumes, suggesting a potential link between increased radiation volume and a higher risk of upper GI toxicity (Supplementary Figure 4).

A chi-square test was conducted to evaluate the association between these subgroups and upper GI toxicity grade. The results indicated no statistically significant association (*χ*^2^ = 6.963, df = 6, *p* = 0.324). The Likelihood Ratio also confirmed the absence of a significant relationship (*p* = 0.250). Additionally, the Linear-by-Linear Association test showed no significant trend across these groups (*p* = 0.305).

Spearman's correlation analysis was performed to investigate the relationship between acute upper GI toxicity and stomach DVH parameters. Among the parameters, D30 cc of the stomach (the volume receiving a 30 Gy dose) showed the strongest correlation with upper GI toxicity (*r* = 0.505, *p* < 0.001), followed by D60 cc (*r* = 0.496, *p* < 0.001) and D10 cc (*r* = 0.483, *p* < 0.001). These results indicate that D30 cc is the most strongly associated with acute upper GI toxicity. However, all three parameters D10, D30 and D60 cc are significant predictors of increased toxicity risk.

**Supplementary Figure 2.**
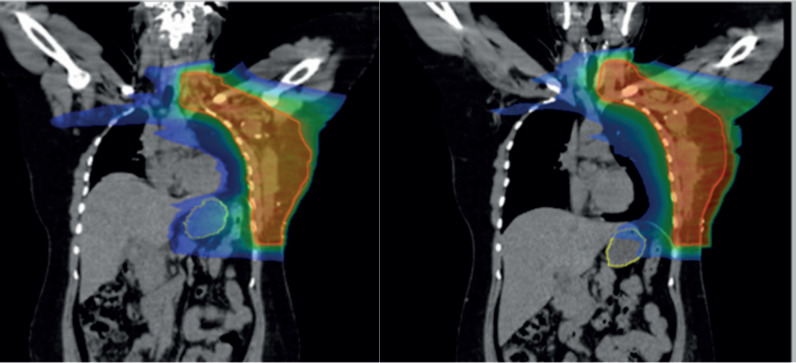
(a): Coronal view showing stomach position in FB (VMAT), (b): In DIBH (VMAT).

**Supplementary Figure 3.**
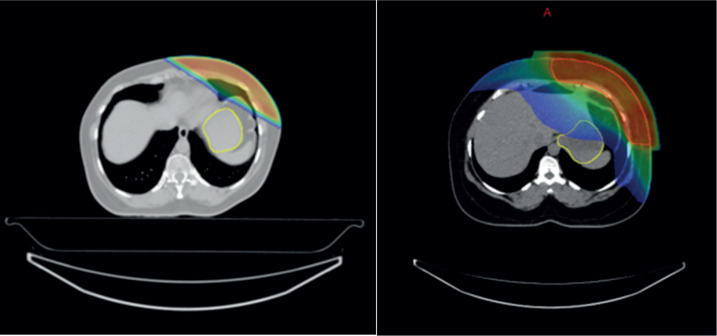
(a and b): Isodose curves showing dose distribution between two RT techniques 3DCRT and IMRT.

**Supplementary Figure 4.**
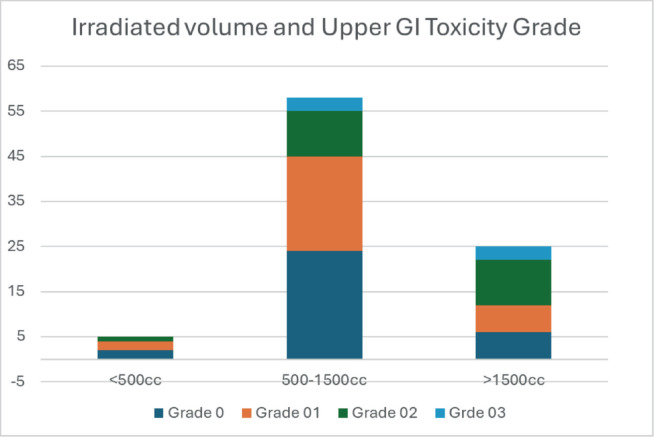
Breast volume categories and distribution of upper GI toxicity grade.

The Mann-Whitney *U* test was used to compare stomach dose parameters (D10, D30 and D60 cc) between FB and DIBH. Stomach D10 cc, the higher-dose region most closely associated with acute toxicity, showed no significant difference between FB and DIBH (Mann-Whitney *U* = 898.500, *Z* = −1.712, *p* = 0.087). This suggests that DIBH helps control the dose to critical stomach sub-volumes, potentially reducing the risk of upper GI toxicity. Interestingly, D30 and D60 cc doses were higher in the DIBH group (*p* = 0.013 and *p* = 0.002, respectively). However, these differences are likely to reflect anatomical and positional variations associated with breath-hold techniques rather than an increased clinical risk. Clinical correlation with upper GI toxicity indicated a protective trend for DIBH, reinforcing its role in mitigating toxicity by improving treatment reproducibility and reducing motion-related dose variability (Supplementary Figure 2a and b).

**Supplementary Figure 5.**
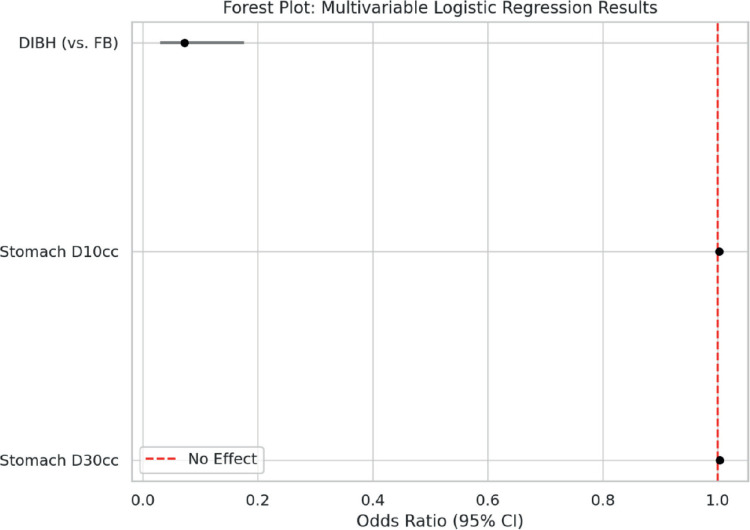
Forest plot showing multivariable logistic regression analysis.

**Supplementary Figure 6.**
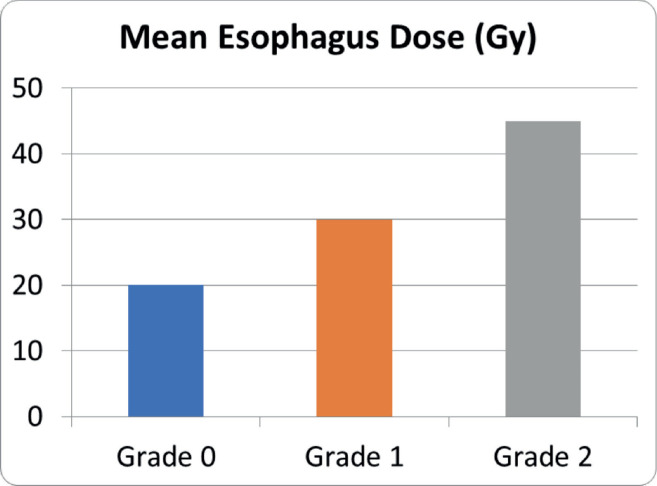
Mean esophagus dose and dysphagia severity.

To address confounding factors and further evaluate this correlation, a stepwise multivariable logistic regression analysis was performed (Table 5 and Supplementary Figure 5). Stomach D30 cc emerged as the strongest predictor of high-grade upper GI toxicity (Grades 2 and 3) (*p* < 0.001), with each 1 cGy increase associated with a 0.3% higher risk of toxicity. Although stomach D10 initially showed significance (*p* < 0.001), its effect was no longer significant after adjusting for Stomach D30 (*p* = 0.411), suggesting potential collinearity between these variables. Notably, DIBH independently reduced the odds of high-grade toxicity by 93% compared to FB (*p* < 0.001), even after controlling for stomach dose parameters (Supplementary Figure 6).

This model accounted for key confounders such as breathing techniques, stomach dose metrics, age and stage, confirming that DIBH provides a protective effect by reducing stomach exposure.

## Correlation between acute dysphagia and radiation treatment characteristics

Dysphagia was a common side effect and was reported in 77 patients, with Grade 1 dysphagia being the most frequently seen in 47 patients (41.8%), followed by Grade 0 seen in 34 patients (30.9%) and Grade 2 seen in 30 patients (27.3%).

Univariate analysis revealed several significant associations between dysphagia grade and clinical factors (Table 6). Surgical extent was significantly associated with dysphagia severity (*p* = 0.019), with higher dysphagia grades observed in patients undergoing more extensive surgeries, such as mastectomy with axillary node dissection. Radiation dose was another significant factor (*p* = 0.018), with a linear trend (*p* = 0.012) showing increased dysphagia severity at higher doses. Patients receiving 4,256 cGy + boost were at the highest risk of developing Grade 2 dysphagia. Breathing technique showed a strong association with dysphagia grade (*p* < 0.001). Moderate dysphagia (Grade 2) was more common in patients treated with DIBH (56.7%) compared to FB (43.3%), while most patients with no dysphagia (85.3%) were treated with FB. Radiation techniques also significantly impacted dysphagia severity (*p* < 0.001). Grade 02 dysphagia was most frequent in patients receiving VMAT (80.0%), followed by IMRT (13.3%), whereas no dysphagia (Grade 0) was predominantly observed in 3DCRT patients (64.7%). BMI was significantly associated with dysphagia severity (*p* = 0.025), with obese patients accounting for 66.7% of Grade 2 cases. Although patients with larger breast volumes (≥1,000 cc) seemed more prone to higher dysphagia grades, the association did not reach statistical significance (*p* = 0.106). However, the likelihood ratio test (*p* = 0.014) suggested a possible trend.

The Kruskal-Wallis test revealed a statistically significant difference in the esophagus mean dose across different dysphagia grades (*p* = 0.000), indicating that the mean esophageal dose is associated with dysphagia severity. This suggests a dose-dependent relationship, with higher mean doses likely contributing to severe dysphagia. These findings highlight the importance of optimising esophageal dose constraints during treatment planning to reduce the risk of radiation-induced dysphagia.

The Mann-Whitney *U* test was performed to assess the clinical relevance of esophageal dose in predicting dysphagia severity. Patients with higher dysphagia grades (Grade 2) had significantly higher esophagus mean doses compared to those with lower grades (*p* < 0.001).

Multivariate ordinal logistic regression analysis was conducted to identify independent predictors of dysphagia severity. Radiation dose (*p* = 0.050) and esophageal mean dose (*p* = 0.002) were significant predictors, indicating that higher radiation and esophageal doses were associated with increased dysphagia severity. Breast volume did not show a significant association (*p* = 0.844). Among the clinical factors, radiation technique was a significant predictor of dysphagia. Patients treated with 3DCRT had a significantly lower risk of dysphagia compared to VMAT (*p* = 0.011). Surgical extent, breathing technique (FB versus DIBH) and other radiation techniques were not significant predictors in the multivariate analysis (*p* > 0.05). These findings highlight the critical role of dosimetric parameters, particularly esophageal dose, in predicting dysphagia severity and emphasise the importance of dose optimisation to minimise toxicity.

## Discussion

This study presents a comprehensive analysis of acute upper GI toxicity in breast cancer patients receiving RT, identifying key clinical and dosimetric predictors. Although radiation to the breast and extremities is traditionally associated with minimal emetogenic risk (estimated <30% in MASCC/European Society for Medical Oncology guidelines), but these data antedated IMRT. Low-dose exposure related to VMAT is a well-recognised phenomenon and its impact on RINV has been reported. The underlying pathophysiology of RINV involves injury to enterochromaffin cells in the GI mucosa leading to release of serotonin (5-HT), this serotonin binds to 5-HT3 receptors on vagal afferents transmitting emetogenic signals to Brainstem’s vomiting centre via area postrema. While the breast and chestwall themselves are not typically associated with high emetic potential, the proximity of stomach to lower edge of the radiation field particularly in left-sided treatments with RNI may trigger this cascade [9].

Over the years DIBH Technique has proven to be effective against cardiotoxicity in left breast/chest wall irradiation with a dosimetric advantage on doses to heart, lung and left anterior descending [16]. In such patients subjected to a radiation field incorporating left sided breast/chest wall with RNI, at times stomach lies within radiation field. With gastric injury during radiation therapy characterised when the upper part of the stomach lies within the radiation field subjecting patients to symptoms such as nausea, vomiting, dyspepsia and abdominal pain reported in around 50% of cases [17]. Later symptoms of radiation-induced gastric toxicity include chronic dyspepsia and abdominal pain due to chronic ulceration from mucosal injury. The estimated radiation dose associated with a 5% and 50% risk of delayed gastric ulceration or perforation at 5 years (TD5/5 and TD50/5) is 50 and 65 Gy, respectively. While specific dose-volume constraints for partial gastric irradiation are lacking, whole-stomach irradiation exceeding 45 Gy has been linked to an ulceration risk of 5%–7% [17]. Most radiation-associated toxicities in breast RT has not regarded RINV as a significant risk factor. However, this study presents comprehensive findings on the correlation between radiation treatment characteristics and acute upper GI toxicity in breast cancer patients undergoing RT. We observed significant associations between certain dosimetric parameters, radiation techniques and the incidence of upper GI toxicity, with several noteworthy results that can transform clinical practice and future research.

In our clinical practice, 26.3% of patients experienced Grade 1 upper GI toxicity, 30% had Grade 2 and 3.6% developed severe Grade 3 toxicity. The correlation between upper GI toxicity and radiation treatment characteristics highlighted that FB was significantly associated with higher toxicity compared to DIBH (*p* = 0.035). These findings can be backed by another study by Yang *et al* [11] reporting significant reduction in upper GI toxicity for patients undergoing adjuvant radiation in DIBH technique (gastric complication rate 4%) versus FB (17.6%) complication rate with a *p* value of 0.026 [11].

These findings reinforce the protective role of DIBH, which minimises dose exposure to the stomach by reducing motion-related variability and improving treatment reproducibility due to physiological changes occurring during DIBH technique leading to expansion in lung volumes and displacement of diaphragm in downward direction leading to lower iso dose curves on stomach, hence reduced toxicity.

Evidence of RT-induced gastric mucosal damage has primarily been documented in case reports involving radiation therapy for malignancies such as lung cancer and GI tumours. Das *et al* [18] demonstrated that the absolute and percentage volumes of stomach receiving 40 and 50 Gy were significantly associated with gastric bleeding in patients receiving RT for intrahepatic cholangiocarcinoma. Similarly, Liu *et al* [19] reported that the volume percentage of intrathoracic stomach receiving 50 Gy strongly correlated with the incidence of Grade 2 and above acute toxicities in patients treated for thoracic oesophageal malignancies. Feng *et al* [20] reported 14% incidence of gastric bleeding post external beam RT (EBRT) at a median duration of 4 months with predicted gastric bleeding risk at TD50 = 56 Gy. This pivotal data highlights the importance of considering the stomach as an OAR to minimise gastric toxicities in breast RT as well. In view of this Yang *et al* [11] analysed dosimetric parameters to stomach in breast RT with radiation dose to 10, 30 and 60 cc of stomach tissue being significantly higher in patients with reported acute GI complications (*p* value: <0.01) [11]. However, in our study a notable observation was the strong positive correlation between stomach DVH parameters and upper GI toxicity. Stomach D30 cc was identified as the strongest predictor of high-grade upper GI toxicity (*p* < 0.001), followed by D60 and D10 cc, indicating that increasing stomach dose is a significant risk factor for acute toxicity. These findings align with previously suggested DVH parameter; however, our study favoring D30 cc as the key predictor of acute upper GI toxicity.

In 1991, Emami *et al* [21] established dose estimates associated with a 5% risk of late stomach toxicities at 5 years (TD5/5), which have since served as widely accepted dose limit guidelines The TD5/5 for severe gastric complications was reported as 50 Gy for whole-stomach irradiation and 60 Gy for one-third of the stomach volume, providing a critical reference for minimising the risk of late toxicities in clinical practice. However, these dose limits are mentioned for 3DCRT era. With the advent of newer techniques like IMRT and VMAT, new dose recommendations for stomach as an OAR is mandated. In our analysis, VMAT was associated with a higher risk of upper GI toxicity compared to 3DCRT (*p* = 0.000), likely due to the more complex dose distribution patterns inherent to arc therapy and low dose spill. However, no significant difference was found between IMRT and VMAT (*p* = 0.083), indicating a similar toxicity profile for these two advanced techniques. Lazzari *et al* [10] demonstrated this phenomenon of low-dose bath exposure secondary to VMAT technique in breast radiation signifying 64% of patients experiencing RINV, which was correlated with DVH parameters of Dmax >10G y and Dmean >3 Gy at gastroesophageal junction and coeliac plexus. These findings highlight that VMAT, while improving dose conformity may result in higher doses to non-quantified structures in particular upper gastro intestinal structures increasing the likelihood of GI toxicity [10].

Our study also demonstrated that higher breast volume was associated with increased upper GI toxicity, with larger volumes (>500 cc) showing a higher incidence of Grades 2 and 3 toxicities. Although the chi-square test did not show statistical significance (*p* = 0.324), the observed trend suggests that breast volume could be a contributing factor, warranting further investigation. Subgroup analysis comparing prescribed RT doses (with and without a boost) showed no significant difference in upper GI toxicity grades (*p* = 0.153), suggesting that the total dose may not be an independent predictor once stomach dose-volume parameters are considered. Our findings contrast with previously published literature that identifies PTV size and delivered dose as significant predictors of toxicity. For patients with PTV ≤700 cc, a dose of 56 Gy was delivered safely without a notable increase in adverse events.

However, for PTV >700 cc, reducing the dose to 50 Gy significantly lowered the risk of toxicity. Multivariate analysis revealed an odds ratio of 2.47 (95% CI: 1.1–5.61; *p* = 0.030), indicating a 2.5-fold increased likelihood of adverse events when the dose exceeded 50 Gy [10].

Our study demonstrated that dysphagia was a common acute toxicity, affecting 70% of patients, with 27.3% experiencing moderate symptoms (Grade 2) requiring medical management. Univariate analysis identified surgical extent, radiation dose, breathing technique, radiation technique and BMI as significant predictors of dysphagia severity. Notably, VMAT was strongly associated with Grade 2 dysphagia (80% of cases), while 3DCRT was predominantly linked with no dysphagia (Grade 0). These findings align with those of a recent study evaluating RE in breast cancer patients undergoing postmastectomy hypofractionated RNI. In that study, Grade 2 and 3 RE occurred in 40.9% and 0.3% of patients, respectively, with no Grades 4 or 5 toxicity reported [21, 22]. The significant association between esophagus mean dose and dysphagia grade (*p* < 0.001) supports a dose-dependent relationship, with higher esophageal doses contributing to more severe dysphagia. Multivariate regression confirmed esophageal mean dose as an independent predictor of dysphagia severity (*p* = 0.002), emphasising the need for stringent dose constraints during treatment planning. While larger breast volumes appeared to correlate with higher dysphagia grades, the lack of statistical significance (*p* = 0.106) suggests that further studies are needed to validate this association. Interestingly, while DIBH reduced upper GI toxicity, it was associated with a higher incidence of moderate dysphagia (*p* < 0.001). This paradoxical finding may reflect differences in dose distribution patterns in the esophagus during breath-hold, necessitating optimised planning to balance the benefits and risks of DIBH.

Our findings have several clinical implications. Firstly, stomach dose-volume metrics (D10, D30 and D60 cc) should be carefully considered during treatment planning to mitigate upper GI toxicity. Among these, stomach D30 cc serves as a particularly reliable predictor and should be prioritised for dose reduction. Secondly, limited literature exists on stomach dose constraints in breast EBRT, highlighting the need for further research in this area. Similarly, minimising esophageal mean dose can significantly reduce the risk of dysphagia, our findings highlight esophagus as a relevant OAR in breast cancer RT, particularly in patients receiving RNI. The significant association between esophageal mean dose and dysphagia severity highlights the need for its inclusion in treatment planning and dose constraint protocols. Thirdly, the observed differences between radiation techniques suggest that VMAT, despite its dosimetric advantages, may require additional caution and dose optimisation to limit toxicity. DIBH remains an effective strategy to reduce GI toxicity. Personalised treatment approaches that incorporate breast volume, anatomical variations and breathing techniques are crucial for optimising outcomes while minimising toxicity. Based on our findings, developing a nomogram incorporating key predictors such as stomach D30 cc, esophageal mean dose, breast volume and radiation technique could help guide individualised treatment planning and improve acute upper GI toxicity grade.

This study is not without limitations. The retrospective design and single-institution cohort may limit generalisability, and the absence of long-term follow-up precludes assessment of late toxicity. Subsequent follow up and longitudinal monitoring can be planned for late toxicity. Moreover, multicenter validation studies are warranted to ensure broader applicability. Hence, the comprehensive analysis of treatment-related factors provides a robust foundation for future research.

## Conclusion

Radiation technique, dose-volume parameters and breathing technique significantly influence the risk of acute upper GI toxicity and dysphagia. DIBH offers a protective effect against GI toxicity, while careful dose optimisation and appropriate technique selection can reduce the risk of dysphagia. These findings support the integration of contouring stomach as an OAR with individualised planning strategies to improve tolerability and outcomes for breast cancer patients undergoing RT.

## Conflicts of interest

None.

## Funding

No funds, grants or other support were received for this manuscript.

## Figures and Tables

**Table 1.** Characteristics of overall study cohort.

Variable	*n* (%)
Mean age	53.03
Histopathological diagnosis	
Invasive ductal carcinoma	93 (79.4%)
Grade I	12 (10.9%)
Grade II	59 (53.4%)
Grade III	22 (20.6%)
Invasive lobular carcinoma	4 (3.6%)
Grade I	00
Grade II	3 (2.7%)
Grade III	1 (0.9%)
Invasive mucinous carcinoma	3 (2.7%)
Metaplastic breast cancer	6 (5.4%)
Neuroendocrine	3 (2.7%)
*T* stage	
T1	15 (13.6%)
T2	30 (27.2 %)
T3	30 (27.2%)
T4	34 (30.9%)
*N* stage	
N0	31 (28.1%)
N1, N2, N3	79 (71.8%)
Mean BMI	
>30 (Obese)	56.9 (51.8%)
25–29.9 (Overweight)	33.99 (30.9%)
18.5–24.9 (Normal)	19.03 (17.3%)
<18.5 (Underweight)	00
Hormone receptor status	
Negative	36 (32.7%)
Positive	74 (67.2%)
HER 2	
Negative	43 (39%)
Positive	67 (60.9%)
Type of surgery	
Breast conserving surgery	41 (37.2 %)
Mastectomy	69 (62.7%)
Radiation technique	
3DCRT	37 (33.6 %)
IMRT	9 (8.1%)
VMAT	63 (57.2%)
Fractionation regimen	
Moderate hypofractionation 4,256 cGy	73 (66.3%)
Moderate hypofractionation 4,005 cGy	33 (30%)
Ultra hypofractionation 2,600 cGy	6 (5.4%)
Lumpectomy cavity boost 1,000 cGy	18 (16.3 %)
Scar boost 1,000 cGy	43 (39.09%)
FB	77(70 %)
DIBH	33 (30%)
Radiation laterality	
Right	43 (39.09%)
Left	67 (60.9%)
Radiation sites	
Chest wall, axilla, supraclavicular region	68 (61.8%)
Breast, axilla supraclavicular region	16 (14.5%)
Chest wall alone	4 (3.6%)
Breast alone	22 (20%)
Chemotherapy	
Neo adjuvant	56 (50.9%)
Adjuvant	54 (49%)
Hormonal therapy	
Yes	67 (60.9%)
No	43 (39%)
RTOG dysphagia grade	
Grade 0: None	34 (30.9%)
Grade 1: Mild dysphagia or odynophagia, may require soft diet	47 (42.7%)
Grade 2: Moderate dysphagia or odynophagia may require liquid diet	30 (27.2%)
Grade 3: Severe dysphagia or odynophagia with dehydration or weight loss >15%	00
RTOG upper GI toxicity scale	
Grade 0: None	44 (40%)
Grade 1: Anorexia, nausea and vomiting not requiring anti emetics, <5% weight loss	29 (26.26%)
Grade 2: Anorexia, weight loss <15%, nausea, vomiting and abdominal pain requiring anti emetics	33 (30%)
Grade 3: Anorexia with weight loss >15%, nausea and vomiting requiring parenteral support, severe abdominal pain despite medications, hematemesis or melena	4 (3.6%)
Key 3DCRT IMRT VMAT cGy: centigray FB DIBH	

**Table 2.** Upper GI toxicity in FB/DIBH cohort.

Toxicity grade	FB cohort (*n* = 77)	DIBH cohort (*n* = 33)	Total (*n* = 110)
Grade 0	29 (26.3%)	15 (13.6%)	44 (40%)
Grade 1	20 (25%)	9 (30%)	29 (26.3%)
Grade 2	25 (31.3%)	8 (26.7%)	33 (30%)
Grade 3	3 (3.8%)	1 (3.3%)	4 (3.6%)

**Table 3.** Univariate analysis for factors associated with acute upper GI toxicity.

Variable	*p*-value
Hormonal receptor status	0.18
Type of surgery	0.072
BMI	1.00
Stomach volume irradiated	0.8
Radiation technique	**0.010**
Lumpectomy cavity boost/chest wall scar boost)	**0.026**

**Table 4.** Subgroup analysis; radiation technique versus upper GI toxicity.

Comparison	Mean difference	*p*-value	Interpretation
3DCRT versus VMAT	−0.815	**0.000**	VMAT associated with higher toxicity
3DCRT versus IMRT	0.015	0.882	No significant difference
VMAT versus IMRT	0.062	0.083	No significant difference

**Table 5.** Multivariable logistic regression analysis.

Variable	Coefficient (B)	*p*-value	Odds ratio (Exp(B))	95% CI for Exp(B)
Stomach D30 Dose	0.003	**<0.001**	1.003	1.002–1.004
Stomach D10 Dose	0.002	0.411	1.002	0.999–1.004
DIBH (versus FB)	−2.623	**<0.001**	0.073	0.024–0.224

**Table 6.** Univariate analysis between dysphagia and clinical factors.

Clinical factor	Test statistic	*p*-value
Surgical extent	*χ*^2^ = 21.379	0.019
Radiation dose	*χ*^2^ = 18.469	0.018
Breathing technique	χ^2^ = 21.206	< 0.001
Radiation technique	χ^2^ = 26.374	< 0.001
BMI	*χ*^2^ = 11.113	0.025
Breast volume	*χ*^2^ = 9.741	0.106

**Supplementary Figure 1.**
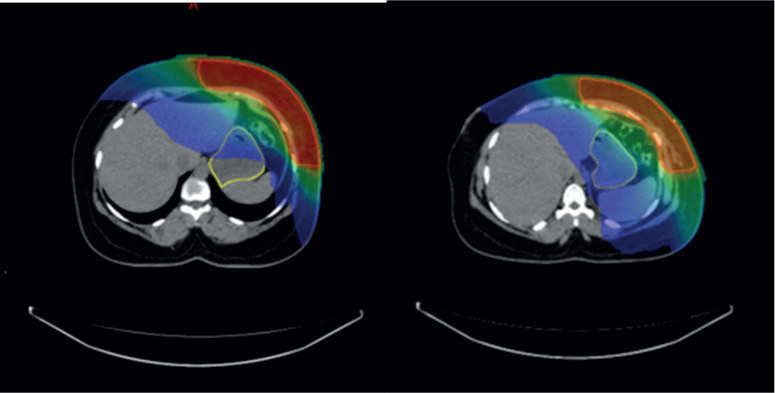
(a and b): Axial CT scan showing stomach position in FB (VMAT Technique), Figure 3b: showing stomach position in DIBH (VMAT Technique).
